# The gift of love: A contemporary view of love in end-of-life nursing care

**DOI:** 10.1177/09697330261424352

**Published:** 2026-03-11

**Authors:** Peter Stuart

**Affiliations:** 1 School of Health and Care, 2706 Coventry University - Coventry Campus, Coventry, UK

**Keywords:** Palliative care, qualitative research, love, care ethics, older persons

## Abstract

**Background:**

Historically love in nursing has been expressed as tender loving care but a move to technological thinking in nursing and the integration of healthcare systems may have changed this to one where love may be distant or avoided from the arena of care. It may now uncertain if love in nursing is an essential part of care, or supererogatory and additional to a paid duty of care.

**Research aim:**

A study was conducted to investigate hospital nurses’ experiences of providing end-of-life care. A core theme from the study was the nurse’s expression of love. This article reports on this outcome and aims to provide clarity regarding the current nature of love in nurses’ care.

**Research design:**

Interpretative phenomenology was used to explore hospital nurses’ experiences of providing end-of-life care.

**Participants and research context:**

6 UK registered nurses with experience of hospital end-of-life care took part.

**Ethical considerations:**

Ethical approval was gained before commencing the study. All participants consented to take part.

**Findings:**

The nurses’ end-of-life care actions were done willingly with goodness, selflessness, sometime courage, placing others before themselves and going beyond their duty of care. As a consequence, their love is universal and not bound by a professional care ethic but given freely of themselves as a gift.

**Discussion:**

The nurses’ action described love as both agape and eros suggesting a universal love. This was suggested a love not bound by a professional care ethic but one given freely of themselves as a gift.

**Conclusions:**

A contemporary view of love in nursing care is presented; when caring for dying vulnerable people in hospital, the value of love for another person is prevalent in the nursing care provided. It is not bound by a duty to care but is an addition to the process of care and is given freely of self.

## Introduction

Love in nursing is explained as unselfishness that involves the responsibility for another’s wellbeing, a selfless commitment to the needs of others.^1,2^ Fitzgerald and VanHooft^3^ conclude that love in nursing requires ‘a willingness, commitment and intention to place the good of the other before the self without reciprocity’. Love is different to nursing care, requiring a dimension of commitment and dedication and the nurse to give something of themselves.

Love may be the secret to providing high quality healthcare.^4^ It requires an emotional investment from the carer, beyond providing technically competent care and is a transformative element of the therapeutic relationship.^5^ Without love, the person can feel uncared for without the inner combination of head and hand and heart the nurse is not present.^6^ As the dates of some of the references will show, love in nursing is not a contemporary subject but as this study demonstrates, is an important feature of current end-of life care experiences of hospital nurses. Love in the context of nursing care needs re-contextualising to a more modern understanding of this declining concept in nursing care.

## Background

Nightingale^7^ in Notes in Nursing objects to the idea that a nurse is someone disappointed in love, but instead is someone who has a ‘love for our kind’ implying an altruistic view of love and nursing care. A century later, Troy^8^ was the first to describe love in the context of the term Tender Loving Care (TLC). Troy defines nursing love in this context as an indefinable trait, present in a nurse’s application of the knowledge of the disease, an awareness of human needs and behaviour, and recognition of the person as an individual. In 1949, an anonymous author recorded a nurse explaining the order ‘TLC’ and explained a patient due to have a serious operation the following day was to have a bath with ‘TLC’; a nice bath with attention to all the details.^9^ The bath in itself was not so important but would help the person to feel important and have confidence in herself and the hospital.^9^ These descriptions suggest the act of loving care was prescribed and not the usual care a patient would receive. This differs to a later view represented by Geissler^10^ study of the expressions and meaning of nurturance in nursing with the finding the nurse appreciates the needs and feelings of her patients and renders loving care; ‘compassionately and non-judgmentally regardless of the patients’ behaviours, situation of beliefs’. Geissler’s study describes nurses’ actions of touch and the sharing of feelings to let the patient know they are safe in their care. TLC is part of the nurturing behaviour of the nurse which Geissler^10^ identifies as fundamental to nursing. This is supported by Kendrick and Robinson^11^ who argue TLC is the ‘core purpose and essence of caring engagement with the patient’ and this is a common theme to descriptions of TLC in nursing. This view contrasts to Fitzgerald and van Hooft^3^ who suggest love in nursing is not asked for, but given freely, outside the role of which one is paid for and is more than the minimum standards, or duty, of care. As a result, they propose love is not implicit in nursing care but something qualitatively different, taking nurses beyond caring to a ‘supererogatory, dimension of commitment and dedication’. Similarly SamsII et al.,^4^ by exploring what physicians and nurses think it means to love their patient, found love encompasses qualities beyond caring, including attributes of sacrifice, trust and honesty. Smith^12^ ^(pg13)^ explores nurse’s caring as labour and the idea of emotional aspects of care as a commodity and not paid for but may be gifted by the nurse. Overall loving care may be identified as an extra dimension to care, requiring the nurse to give more of themselves.

Despite the historic connection between love and nursing, the term love is disappearing from nursing education and practice.^5,13–15^ Stickley and Freshwater^14^ suggest ‘love’ has been socially minimised and has left a void the professional care’s vocabulary. The exception is the nurse-patient relationship and the therapeutic and healing values embedded in the North American and Scandinavian concept of Caritas Care. This is confirmed by the limited literature recently published on love in nursing which is predominantly north American and Scandinavian in origin. Arman and Rehnsfeldt^13^ suggest resourcing and technological thinking as well as male attitudes have led nurses to fear expressing love. In addition, the healthcare system can be dehumanising, technologically focused and bureaucratic.^4^ This implies love in nursing has moved from an order of tender loving care, where attention would be paid to the person, to one where love may be distant or avoided from the arena of care in an environment of health systems and technicality are more present.

Not all studies found the distant and technical approach to care to be true. Goldin’s^15^ North American study found nurses stressed the importance of connection between the person and the nurse. Getting to know the person and touching the person were seen as expressions of love, concluding nursing must learn to embrace the Caritas concept of love. Caritas is a term used in caring science approach to nursing care to mean cherish, appreciate and give special loving attention to with charity, compassion and generosity of spirit.^1,2,16,17^ Thorkildsen et al.^2^ suggests the idea of Caritas is rooted in Christian ideas of charity, such as the good Samaritan where the core of caring is compassion for a fellow human being derived from meeting a suffering human being. Caritas can also be expressed as agape; neighbour-love, a selfless and ethical love.

The evidence presented is mostly dated and unclear as to the current nature of love in nursing care. In addition, it is uncertain if love is part of nursing care, or supererogatory and additional to a nurse’s paid duty of care. The following article reports on part of a study conducted to investigate hospital nurses’ experiences of providing end-of-life care. A finding from the study was the nurse’s expression of love in their end-of-care. It is this aspect of the study this article focuses on and in doing so the findings help to provide some clarity to the current understanding of the nature of love in nurses’ end-of-life care in the technological and curative focused hospital environment and how this understanding also contributes to informing pre and post registration nursing education and training as well as nursing care leadership and practice.

## Research aim

A study was conducted to understand hospital nurses’ experiences of providing end-of-life care. A core theme from the study was the nurse’s expression of love in their end-of-care and it is this finding this article reports on and aims to provide some clarity regarding the current understanding of the nature of love in nurses’ care.

## Research design

To achieve the overarching aim of the study of exploring hospital nurse’s experiences of providing end-of-life care Interpretative Phenomenology was used as this approach recognises the individuality of the lived experience of the phenomena.^18,19^ While other approaches have similar aims, interpretative phenomenology also values the researcher’s presuppositional knowledge and enables the researcher to interpret, with caution, which can be a valuable guide to inquiry.^18–20^ The researcher had an emic perspective with the interpretation both as a hospital nurse and a specialist nurse in palliative and end-of-life care.

### Methods and data collection

**Figure 1.**
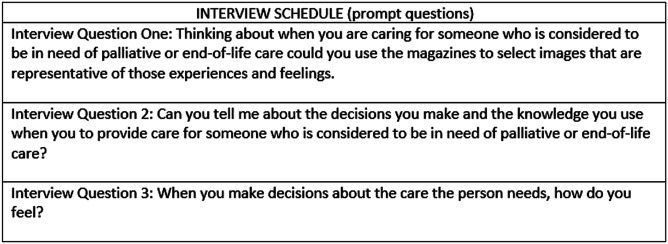
Interview questions.

Reliving end-of-life care experience can be emotional and visual research methods offer ways of exploring and articulating emotion that may be difficult with language-based methods.^21–24^ Collage was used as a method of data collection as this can provide an opportunity for participant to create visual representations of their worlds using pictures.^25^ Participants were asked at the beginning of the interview question 1 (Figure 1) by the researcher to select images that represented their palliative and end-of-life care experiences and to build a collage. The researcher supplied the same range of image rich magazines for participants to create their collage from. Participants were left alone to do this with no time constraint.

Mannay^6^ makes the point that when viewing participant directed images, the interpretation of the audience may not be the same as the image maker. This emphasises the need to provide participants with the opportunity to explain the image. For this reason, an elicitation interview, lasting 45–60 min, was conducted immediately after participants completed the collage by the researcher and audio recorded with field notes to support reflexivity.

### Data analysis

The transcriptions of the interviews were analysed by the researcher following Ricoeur’s hermeneutic approach to analysis. Interpretive, or hermeneutic, phenomenology is more than description of the concepts relevant to the human experience but an exploration of the meanings.^26^ Ricoeur agrees with this view and argues interpretation is needed to make sense of our lifeworld.^27^ This study asked nurses to create visual metaphors as explanations of their end-of-live care experiences. Ricoeur reports on the difficulty of understanding the intentionality of the metaphor is text can be analysed according to syntax, but this provides the reader with an understanding of what the author has said according to the rules of syntax, which provides only a literal understanding.^28^ ^(pg 98–100)^ Ricoeur uses the metaphor ‘man is a wolf’ as an example, this analysed literally as ‘man to be of lupine construction’ and no new meaning comes from this.^28^ ^(pg102)^ Seeing the phrases as metaphor changes the meaning from one of none literal understanding, which places an emphasis on ‘opening up a world’.^28^ ^(pg 110)^

Ricoeur’s offers a theory of interpretation to help understand the world; however, this is more of a philosophical approach than a research method.^27,29–31^ Geanellos, Tan and Hardwick have applied Ricoeur’s theory of interpretation to the analyses of their health focused research texts and present an applied form of Ricoeur’s process of interpretation, which this study follows. The hermeneutic circle Ricoeur argues is an unavoidable structure of interpretation which is a continual process by which the text is seen in relation to its parts and the whole, allowing understanding to be developed.^28,29,31^ ^(pg 107)^ The process begins with an explanatory approach which involves selecting the main details of the text, presenting superficial meanings. This is followed by an initial naïve interpretation which takes at face value the existing understanding, which may be naïve and rejected, or developed and expanded.^29,31,32^ ^(pg180)^ Structural analysis brings out interpretation from the explanation and is a stage ‘between naïve understanding and critical interpretation, between surface understanding and a depth interpretation’.^32^ ^(pg 180)^ Geanellos explains interpretation addresses only that which is within the text, whereas understanding seeks to go beyond what is expressed to the unexpressed, drawing on the interpreters’ preunderstandings. The process opens up the interpretative possibilities, giving weight to opposing arguments and defending, with evidence and reasons.^29,32^ ^(pg 178)^ The process objectifies the text removes the authors intent, allowing interpreters to move away from the idea that the only understanding is that of the research participant.^31^ As a result, there can be multiple interpretations of the text. Riceour’s theory of interpretation achieves objectification through distanciation, which is the freeing of the text and giving it a life of it own.^31^

**Figure 2.**
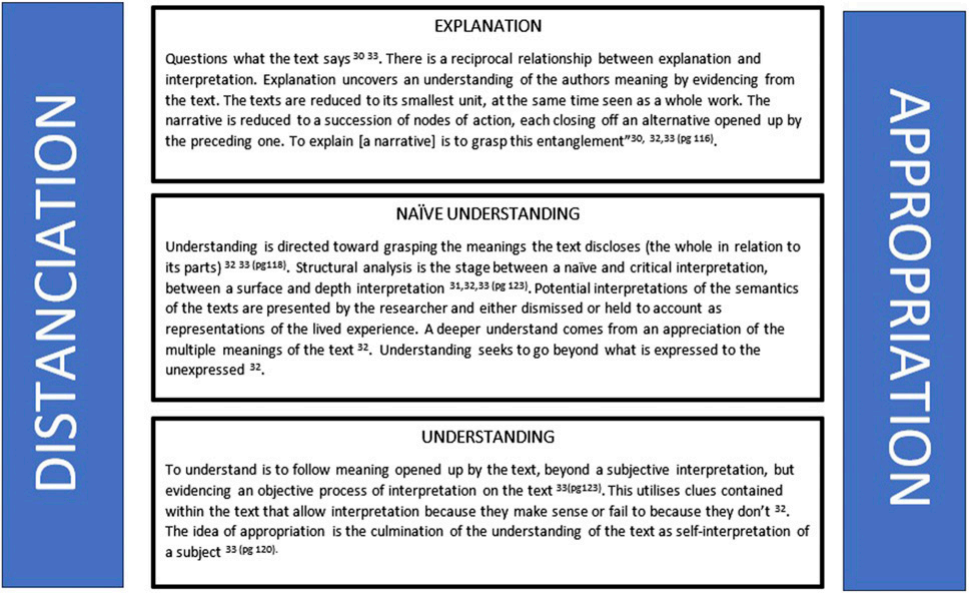
Ricoeur’s structural interpretation.

Appropriation, making ones own, then becomes the focus, rather than on explaining participants unique meaning.^31,32^ This final phase creates new ways of knowing as appropriation and in the context of metaphor new ways of meaning and knowing emerge (Figure 2).^28,30^ ^(pg110)^

### Trustworthiness

Pollit and Beck’s^33^ approach to enhancing quality in qualitative research was followed to maintain trustworthiness. Data collection was conducted over a long period of 18 months, as was data analysis. This prolonged engagement enabled the researcher to focus on the details of the descriptions as well as the emerging whole, aiding saturation of data. The application of interpretation and presupposition in the analysis of the text could threaten trustworthiness but Ricoeur’s structured approach balances the credibility by arbitrating between the possible interpretations.^27^

## Participants and context

**Figure 3.**
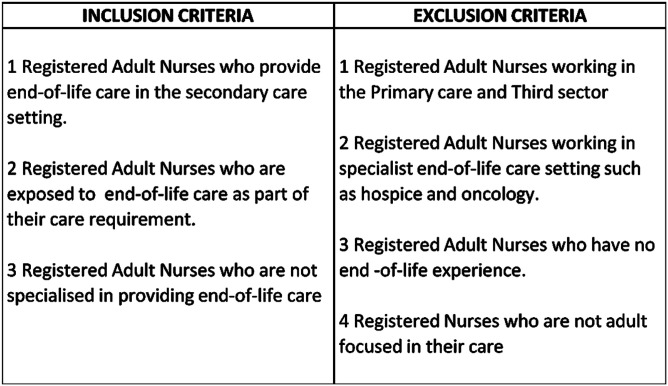
Inclusion and exclusion criteria.

**Figure 4.**
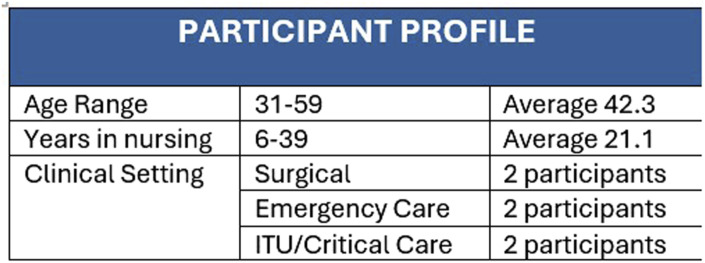
Participant profile.

Participants were recruited from three acute English hospital trusts and in a university using purposive sampling, allowing selection based on their knowledge and experience of the phenomena.^34^ ^(pg353)^ An inclusion and exclusion criteria (Figure 3) were applied to ensure participant held relevant experience. A total of 6 participants were recruited, see profile summary Figure 4. Pseudonyms are used to protect the participants identity.

## Ethical considerations

Ethical approval was gained from a UK University Research Board before the study commenced. Local NHS Research ethics approval was requested but considered as unnecessary as not utilising patients or NHS premises. Written informed consent was acquired from all participants prior to them taking part. Once the recorded interviews had been transcribed, real names and identifying data was changed to pseudonyms to maintain confidentiality. Visual imagery was digitally recorded, identified by the same pseudonym only and stored digitally, with encryption, to support confidentiality.

## Reflexivity

The interpreter of the data had experience as a male ward nurse and as a hospital Specialist Palliative Care nurse. The insider, or emic, position was both valuable and a hindrance during the interpretative phase. Ricoeur’s approach to interpretation requires the interpreter to draw on their preunderstanding to go beyond what is expressed to the unexpressed. Despite the process the interpreter initially avoided seeing love as being present within the results, as this did not sit comfortably with their own experience. However, by applying Ricoeur’s method of distanciation, the mediating of the text and self-understanding, the interpreter recognised the experiences of the participants was similar to their own and was in keeping expressions of love.

## Findings

### Hospital nurses’ expression of love in end-of-life care

The nurse’s metaphors described end-of-life care actions that were done willingly with goodness, selflessness, placing others before themselves and going beyond their duty of care. The actions required the nurses in this study to work beyond the limits of their role, sometime requiring courage, to provide care. They gave of themselves to be able to continue caring and did not avoid end-of-life care. As result they were doing more than their duty by providing end-of-life care principled by love. The consequence is the nurses acts of end-of-life care present love as a core virtue and, although given of themselves was integral to the end-of-life care they gave.

**Figure 5.**
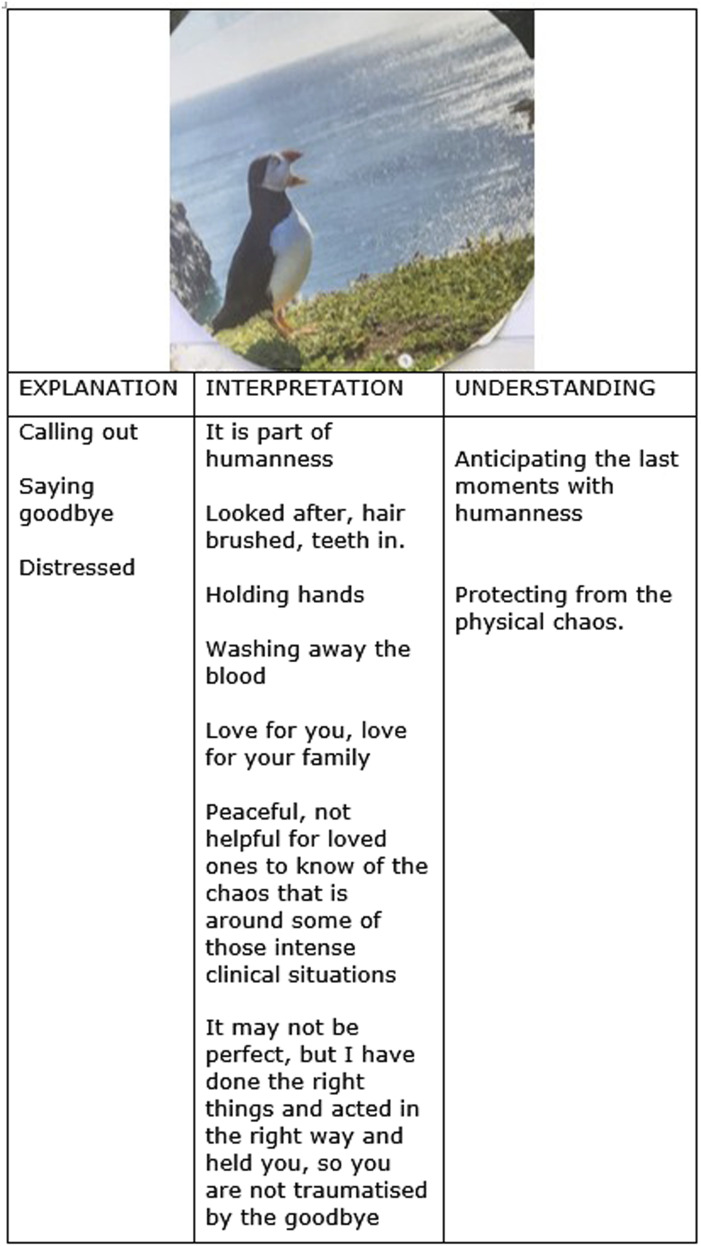
Louisa goodbye.

Louisa describes Saying Goodbye (Figure 5) as her feeling of love for her patients as a nurse and the importance of showing that the person is loved:

For me it’s the value, I want to make you loved, whether that is loved for you, or loved for your family. How I display that, sometimes that is the hand holding, and sometimes it is washing the blood away. It’s the same value. (Louisa)

Louisa’s puffin image is about saying goodbye (Figure 5), representing her experience of preparing families and protecting them from the physical reality. By acting in the right way, washing away the blood and clearing the mess as well as holing the person Louisa’s hope is they are not traumatised by the goodbye. What they need to see is:

dad has been looked after because his hair is brushed, and he has got his teeth in, because they are the bits about care that they know about. Whereby helping them by making your patient comfortable clean and hair brushed actually what you are saying is, its calm and look how well looked after dad has been and loved

Louisa’s metaphor of ‘saying goodbye’ is the anticipation of the last moment of life, meeting it with humanity and a desire to protect her patients from the physical trauma of the ‘goodbye’.

**Figure 6.**
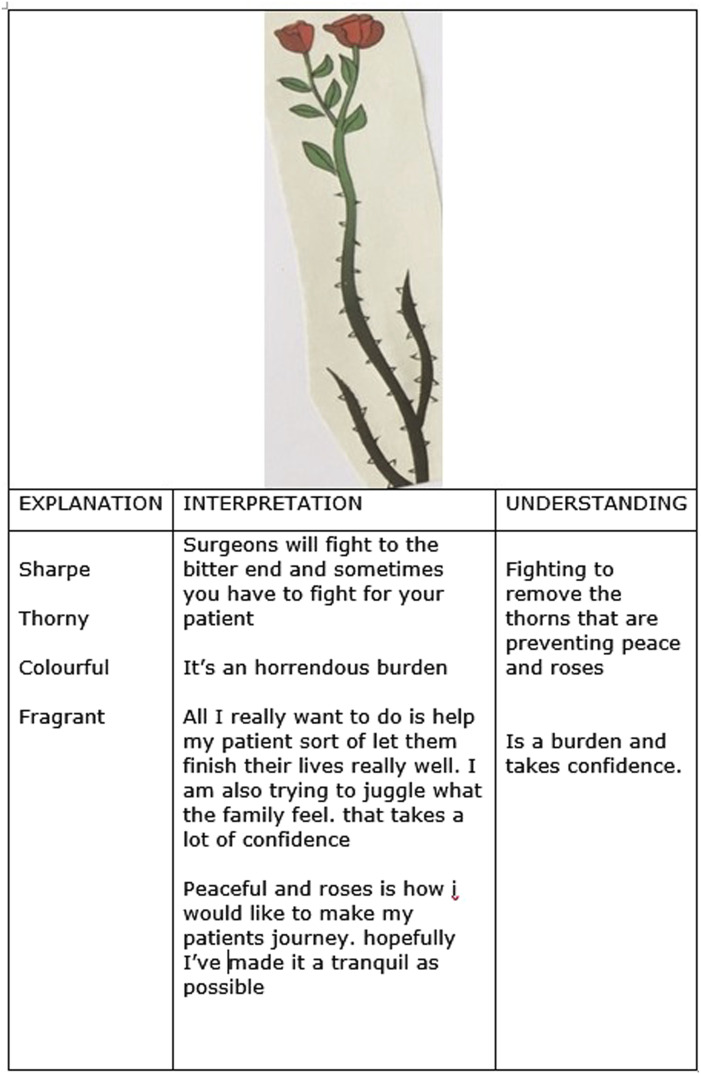
Rose without thorns (Virginia).

Louisa’s values are present in other nurses’ accounts. Working on a surgical unit Virginia’s metaphor of a rose (Figure 6) represents her end-of-life care intentions: ‘A rose without the thorns at the bottom is how I would like to make my patient’s journey’. Virginia describes this further as end-of-life care intentions to remove the thorns by making the process calm and peaceful adding this is not always easy: ‘dare I say it, if you are under the surgeons, still surgeons will fight to the bitter end and sometimes you have to fight for your patient’ identifying the burden this created and courage needed. Virginia’s metaphor of a rose without thorns is having the courage to defend her aim of providing end-of-life care as peace and roses. This requires her to do more than her duty and go beyond the limits of her role.

**Figure 7.**
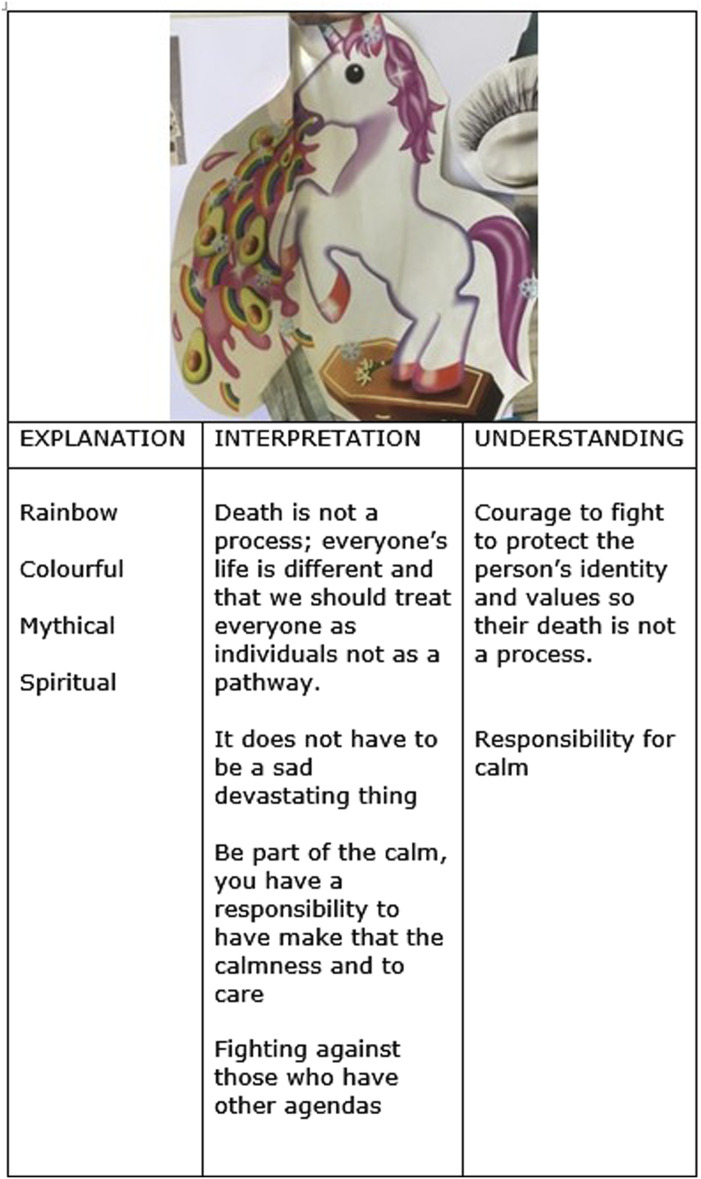
Protection (Mary).

Mary’s metaphor of a Unicorn (Figure 7) is a reminder to Mary that death should not be a process, ‘they had a life and dreams and hope like all of us. Like when they got to the hospital bed about to die’. The Unicorn reminds Mary to protect her patient’s identity and vulnerability, to make dying more personal. This was often difficult in a faced paced environment of A&E that often worked against Mary’s idea of protecting the uniqueness of the individual in end-of-life care:

you are constantly against others who have their own agenda and targets pushing people through. I remind people that patients are human beings. (Mary)

Mary tries to enable end-of-life care to be individual, focussing on the person and the life they have had, protecting their identity, and values. To do this Mary is required to work with authority, taking responsibility to do what she believes is right for the person, to work selflessly against established norms. Mary’s Unicorn metaphor is the courage to protect the individual’s values and identity so their death does not becomes a process. It symbolises Mary’s commitment to safeguard individual identity during challenging times.

**Figure 8.**
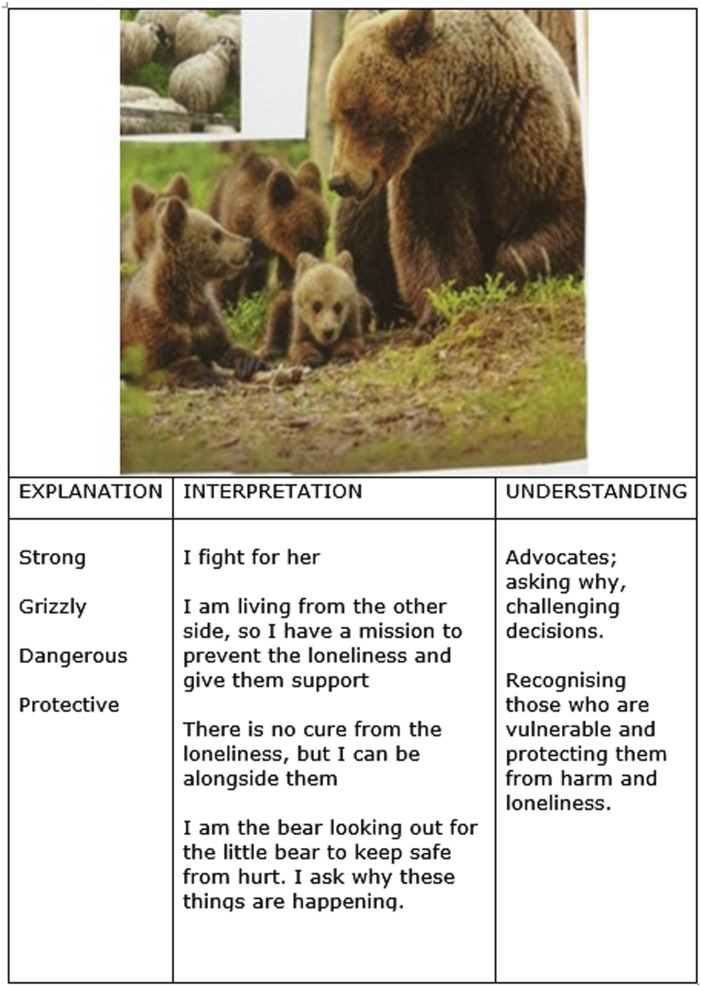
(Martha) bear.

Martha refers to her metaphor of a bear (Figure 8) and how this bear recognises the vulnerability of dying patients and works to protect them. Martha describes the bear as ‘keeping safe from hurt’ and recalls situations where she has questioned and challenged clinical decisions she felt were not right to keep the person safe. Martha also views of human contact as being essential in end-of-life care and Martha’s bear metaphor is indicative of end-of-life care as being more than physical care. It means recognising those who a vulnerable in end-of-life and protecting them from harm and loneliness. As with other nurses in this study, Martha as the bear goes beyond caring and integrates herself as protector, bringing dedication, commitment and a desire to do good into her end-of-life care.

**Figure 9.**
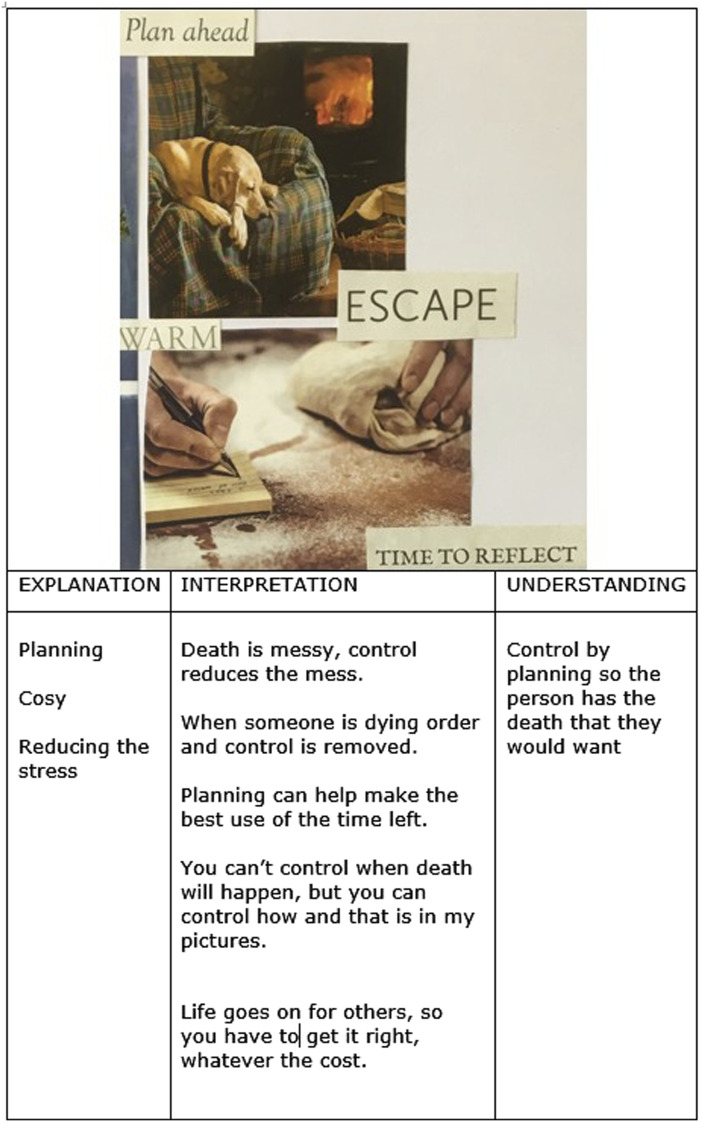
(Hilde) control.

Hilde selected images that represented control (Figure 9). She describes her experience of end-of-life care and death as messy, and she tries to avoid the mess by controlling and carefully planning end-of-life events:

The nature of death, it is a finite thing you can’t change that, so if when all these messy emotions are involved…my theory is by resolving all of the physical bits, then it may be contributing to all of the emotional bits (Hilde)

Hilde describes control as planning and preparing as much as she can and in doing so aims to avoid the mess of death. Hilde does everything she can to prepare for the death; by putting the plans she has discussed with the patient and the family into action and communicating with her team the requests, continually questioning if everything has been done. Hilde describes control as planning ahead so the person has the death they want. In doing so Hilde is similar to Martha by being motivated to protect the patient and their family from the mess of death is based on a desire to good for the patient, as well as a dedication and commitment to the patient.

**Figure 10.**
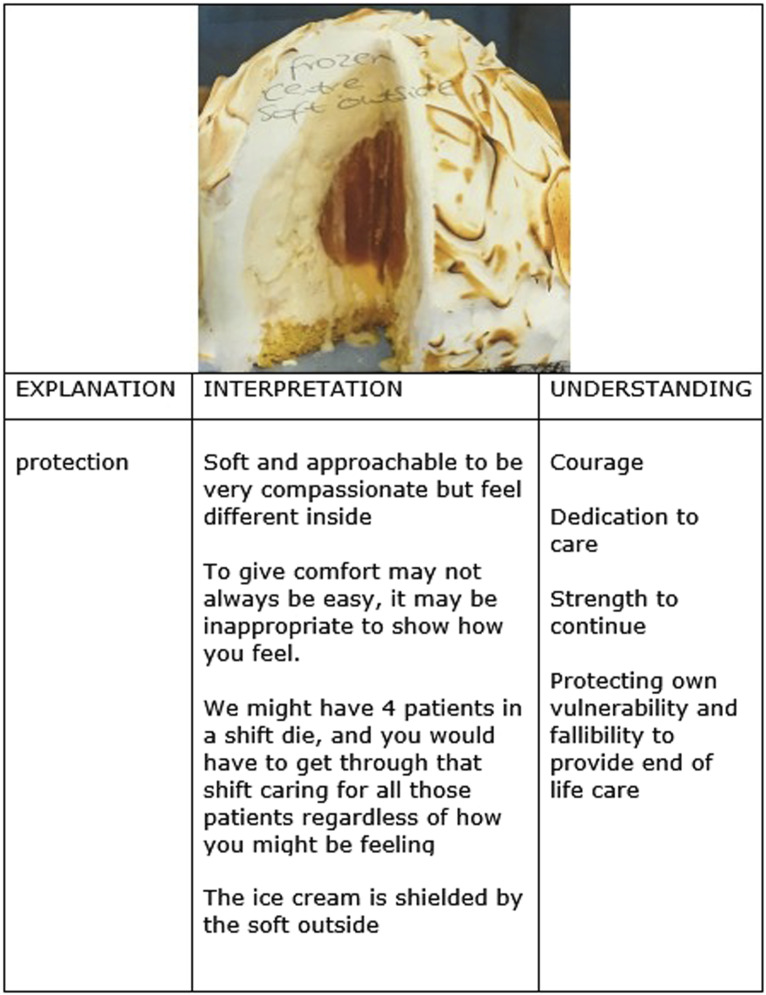
(Nancy) baked Alaska.

Nancy, working in ITU states uses the metaphor of baked Alaska (Figure 10). Nancy describes: ‘I would defy you not to cry where it is an 8-year-old [who has died] and mum has dressed him in his football kit… We might have four patients in a shift die and you would have to get through that shift caring for all those patients regardless of how you might be feeling’. Nancy’s Baked Alaska means having the strength to continue, she is shielding or protecting her own vulnerabilities in order to continue end-of-life care. Such a situation is very challenging, requiring courage and exemplifies similar ones described by other nurses.

Louisa makes the comment ‘I have loved you as much as I can as a nurse’ and recognises she may not have resolved the ‘bubbling secretions and pain’ but ‘I have acted and genuinely and feel I have done all that could be done… to make you look loved’. Nancy and Louisa share a vulnerability and fallibility as well as fidelity and determination.

Common to the nurse’s metaphors is protection from some form of harm or trauma, and a responsibility and willingness to create calm. For some this required courage to achieve. In addition, the nurses metaphors describe a commitment, dedication and giving of themselves. They are expressing loving care for their end-of-life patient as well as a loving connection and dedication.

## Discussion

The nurses, when providing end-of-life care describe actions such as washing away the blood and focussing care on the individual are good nursing care. The nurse’s actions to protect while providing end-of-life care are done with dedication and commitment, and at the same time some of them require acts of courage, selflessness and in some cases admission of vulnerability and fallibility. Viewed in this way the nurses’ actions indicate more than good nursing care and not only is love integral to their end-of-life care but as an additional giving of themselves in that moment.

By providing end-of-life care in this way the nurses’ actions appear congruent with descriptions of neighbour-love, or agape as a benevolent, unconditional universal love given without reciprocity.^35–37^ Levinas argues agape is the ‘selfless love of humanity’ and an ethical love, not one born from sexual desire and carnal enjoyment.^38^ Outka^35^ also defines agape as an ethical principle in relation to neighbour-love but adds that everyone has worth which means agape respects the worth of all human beings equally and not simply one’s own interest.^35^ ^(pg17),36^ ^(pg168)^ Similar to Outka, Tilllich^39^ views agape as not questioning the person being worthy of love, approving of the love, nor is it based on favouritism, it is universal and given unconditionally.^39^ ^(pg280–281)^ In this study when providing end-of-life care the nurses were influenced by their aim was to protect from harm, trauma and mess of death and did not question if the person was worthy of their love and their actions support this view of love as agape, as non-discriminatory.

The combined experiences of the nurses in this study suggest a passion and a caring concern for their end-of-life patients influenced by a desire to do all they possibly can for them and ‘keep safe from harm’. This desire to ‘use’ good has a self-fulfilling relationship and need for personal connection indicative of love as eros.^40^ ^(pg17)^ Levinas describes eros as an erotic love, a love of life and involves egotistical enjoyment of life which can influence the selfless love of the Other.^38^ ^(pg66)^ This is not describing the nurses’ experiences of love when providing end-of-life care. Tillich regards eros as more than erotic and describes eros uniting with epithymia (libido) as desire for self-fulfilment and a love for the beauty found in nature and culture (aesthetics).^37(pg29–30) pg119)^ Socrates in Symposium presents Eros as the son of Penia (Poverty) and Poros (Resource) creating Eros as a mediator between need and resource which gives Eros a unifying characteristic.^41^ ^(pg79)^ Socrates also presents Eros as desiring beauty, not just physical desire, but beauty in learning, observation and in itself; the very essence of beauty.^41^ ^(pg 94)^ This moves eros from a need, a craving a ‘vulgar eros’ to eros as a vitality and a caring concern.^42^ ^(pg5)^ Eros defined in this way, as self-fulfilment, vitality, unifying and a caring concern is more aligned to how the nurses in this study describe their end-of-life care.

Tillich goes further and argues that eros and agape should be united, an idea rejected by others as mixing fire with water.^42,43^ Levinas distinguishes between eros and agape, and insists on their segregation, arguing eros is involved with egotistical enjoyment, and agape is a selfless ethical love.^38^ ^(pg66)^ Tillich^37^ ^(pg119)^ believes agape is a reuniting force of love, bringing together the separated parts to reform in the individual person a self-centred and complete being. Tillich adds philia and argues that he who cannot love the friend (philia) cannot love culture (eros) and agape cuts through friendship and indifference, desire and disgust, and it does not need sympathy to love; agape ‘loves in everybody and through everybody love itself’.^37^ ^(pg119)^ This unification is evident in Hilde’s desire to control the mess of death, Mary’s metaphor of a unicorn and Martha’s bear symbolising protecting the individual at difficult times and circumstances; in Nancy’s and Virginia’s challenging end-of-life care situations possessing the courage and desire to continue caring; in Louisa’s action of washing away the blood, hand holding, she is acting with goodness and expressing her desire to express ‘you are loved’. Both Nancy and Virgina describe the cost of providing end-of-life care as a burden and the need to shield themselves in order to continue. In doing the nurses are evidencing components of love as both agape, philia and eros in their end-of-life care. Their actions of placing others before themselves, going beyond the normal boundaries or duty of care to give of themselves.

Bevis,^44^ ^(pg 55)^ writing some time ago (1988), believes there is a difference between caring and love in nursing, describing in caring both persons must be served and in most interactions, caring is egalitarian. Love is altruistic and self-sacrificing by requiring the giving up of one’s own needs for the benefit or service of another. Despite its age Bevis’s argument is evident in the nurse’s experiences within this study by giving up their own needs in providing end-of-life care to the benefit of another. Fitzgerald and Hooft^3^ also suggest such actions represent love in nursing as qualitatively different from caring and something more than nurse’s ethical stance towards their patients. This study supports this view but adds that when providing end-of-life care the nurses were motivated by a desire to do good, to protect and sometimes requiring courage. Ricoeur argues it is ‘scandalous’ to command love as feeling as an ethical imperative.^45^ ^(pg26)^ The nurses did not describe actions that suggested they were commanded or obligated to provide the end-of-life care they gave, was influenced to protect from harm and create peace and humanness, by love to do good. This indicates an unconditional love which Ricoeur argues such love is hyperethical in that it transcends ethics and is given freely without reciprocity. Ricoeur introduces ‘economy of gift’ in which economy is an exchange and gift is what the giver gives; love.^45^ ^(pg28)^ In keeping with Ricouer’s idea of love as a gift the nurses gave part of themselves. Ricoeur discusses the rule of reciprocity as ‘give because it has been given’^pg 36^ but in this study, the nurses expected nothing in return; they give freely of themselves, which breaks this rule of reciprocity, supporting Ricoeur’s idea that love the nurses gave when providing in end-of-life care is hyperethical or unconditional.

The nurses in this study could have provided technically proficient, evidence-based end-of-life care. Such care can be without love and without regard for human values.^13,46^ Care without love can be altruistic but the emphasis can become focused on evidence-based care, technical skills and bureaucratic demands that objectifies the patient.^14,44,47^ The result, Stickley and Freshwater^14^ argue, is a focus on being professional with practitioners fearing reprisal for their expression of love as being unprofessional. This is not the finding of this study. As the nurses in this study demonstrate when providing end-of-life care love preserves humanity and human dignity.^46^ All the nurses were influenced by their wish to protect their dying patients from what they regarded as the harm, trauma and mess of death. This care required courage and required protection of their Self. For some this was a burden but for all end-of-life care was given freely. As a consequence their love for Other was universal, and evidences that their end-of-life care is superogatory, more than a professional ethical stance, providing love that is unconditional and given as a gift.

## Limitations

Interpretative phenomenology with the absence of epoché is open to the influence and bias of the researcher there is a risk that the researchers concealed meanings, undisclosed power influence in the interviews and self-interest could produce superficial outcomes.^48^ Solutions are offered by providing structured processes to reflect the participants view and not the subjective view of the researcher.^20,34,49^ All the participants were female and therefore any gender variations of the experience of the phenomena cannot be accounted for. In particular emotions and protection of self among male nurses may be different with a suggestion that male nurses feel uncomfortable with emotional contact.^49^ The participants were experienced nurses with between 6 and 39 years in practice. Current evidence suggests length of work experience is positively related to less anxiety about death.^50^ As a result, recently qualified nurses may have different experiences, and this is not accounted for in this study.

## Conclusion

Exploring hospital nurses’ experiences of providing end-of-life care has resulted in a more contemporary awareness of how love fashions nursing end-of-life care. This has been explained as love that combines eros with philia and agape to form a universal love that is egalitarian, good, protective and at times courageous. It is not bound by a profession care ethic as duty to provide but is an addition to the process of care and is given freely of self as a gift. It is suggested that present day nursing care can be technically focused, evidenced based and bureaucratic but what this study identifies is when caring for dying vulnerable people in hospital the value of love for another person is prevalent in the nursing care provided. This should not be seen as unprofessional but recognised as a gift chosen by nurses to be given freely and valued by practitioners, managers, educators and those developing end-of-life services and care policy.
